# Ecrg4 Attenuates the Inflammatory Proliferative Response of Mucosal Epithelial Cells to Infection

**DOI:** 10.1371/journal.pone.0061394

**Published:** 2013-04-23

**Authors:** Arwa Kurabi, Kwang Pak, Xitong Dang, Raul Coimbra, Brian P. Eliceiri, Allen F. Ryan, Andrew Baird

**Affiliations:** 1 Department of Surgery, University of California, San Diego School of Medicine, La Jolla, California, United States of America; 2 Veterans Administration Medical Center, San Diego, California, United States of America; University Hospital Hamburg-Eppendorf, Germany

## Abstract

We report an inverse relationship between expression of the orphan candidate tumor suppressor gene esophageal cancer related gene 4 (Ecrg4), and the mucosal epithelial cell response to infection in the middle ear (ME). First, we found constitutive Ecrg4 mRNA expression in normal, quiescent ME mucosa that was confirmed by immunostainning of mucosal epithelial cells and immunoblotting of tissue lysates for the 14 kDa Ecrg4 protein. Upon experimental ME infection, Ecrg4 gene expression rapidly decreased by over 80%, between 3 to 48 hrs, post infection. When explants of this infected mucosa were placed in culture and transduced with an adenovirus (AD) encoding Ecrg4 gene (ADEcrg4), the proliferative and migratory responses of mucosal cells were significantly inhibited. ADEcrg4 transduction of control explants from uninfected MEs had no effect on basal growth and migration. Over-expression of Ecrg4 *in vivo*, by pre-injecting MEs with ADEcrg4 48 hrs prior to infection, prevented the natural down-regulation of Ecrg4, reduced mucosal proliferation and prevented inflammatory cell infiltration normally observed after infection. Taken together, these data support a hypothesis that Ecrg4 plays a role in coordinating the inflammatory and proliferative response to infection of mucosal epithelium suggesting a possible mechanism for its putative anti-tumor activity.

## Introduction

Mucosal surfaces represent a major barrier lining and protecting the ducts of the eye, ear, exocrine glands and the aero-digestive and uro-genital tracts [1]. In particular, mucosal epithelia serve critical homeostatic functions as biological, physical and mechanical barriers that regulate innate and adaptive immunities and the tissue response to infection and injury [2], [3]. It is generally accepted that the expression of cytokines, chemokines and antimicrobial factors mediate much of the mucosal response to injury [4], [5], [6], [7]. However, the mechanisms that regulate local homeostasis in normal mucosal epithelium and how these mechanisms may participate in response to injury are less understood.

In recent years, the identification of paracrine, juxtacrine and autocrine factors that control the inflammatory response has led to significant refinements in our understanding of tissue homeostasis. Local factors that are constitutively produced in tissues respond to changes in the local milieu to play critical roles in defining ultimate biological responses. For example, constitutively expressed defensins [8], [9] and pathogen receptors [1], [4], [6], [10] encode classes of molecules poised to defend tissues from infection. In a similar manner, the induction of alarmin genes [11], [12] after inflammation is a response to the detection of biological, chemical and physical threats that disrupt tissue homeostasis.

We recently identified a candidate gene called Esophageal cancer related gene-4 (Ecrg4) that we proposed plays a sentinel function to monitor set points of homeostasis [13], [14], [15], [16]. Constitutively expressed by numerous cell types, localized in many normal tissues, and found in selected biological fluids, Ecgr4 is a member of both the secretome [17], [18] and neuropeptidome [13], [14], [19], [20] that is tethered to the epithelial cell surface [13], [14], [15], [16]. In cancer, its expression is epigenetically regulated by DNA methylation of >16 CpG sites in its promoter region [21], [22], [23] and as such, it is highly down-regulated in epithelial cancers via hypermethylation [21], [22], [23], [24], [25], [26]. At 148 residues in length, the Ecrg4 protein is slightly basic (pI 8) and, depending on its posttranslational processing, can generate several potential ligands of 2 to 14 kDa [14].

Because previous studies demonstrated that Ecrg4 gene expression is associated with epithelial cells, cancers and barriers [16], [25], [27], [28], we tested the possibility that Ecrg4 could regulate the inflammatory response to infection. In the studies presented here, we exploited the capacity of ME mucosal epithelia to respond to infection with both proliferation and inflammation. We showed that while Ecrg4 is constitutively expressed in normal epithelial mucosa, it is rapidly down-regulated during bacterially mediated otitis media (OM) and that with its inappropriate expression during infection, it can modulate the natural course of the inflammatory response both *in vitro* and *in vivo*.

## Materials and Methods

### Hyperplastic epithelial middle ear mucosa *in vitro* model

All animal studies were performed in strict accordance to the recommendations in the Guide for the Care and Use of Laboratory Animals of the National Institutes of Health (NIH). All animal studies performed were carried out in strict accordance with an approved UCSD Institutional Animal Care and Use Committee (IACUC) protocol (Protocol no. S08281) specifically approved for this study. All surgeries were performed under anesthesia, and all efforts were made to minimize suffering. The bullae of ∼300 g male Sprague-Dawley rats were bilaterally injected with ∼50 µL saline (control) or saline containing 10^5^ cells/mL *Haemophillus influenzae* strain 3655 (nontypeable Hi/biotype II). Following the inoculation, the tympanic membrane was visually confirmed to be intact. At ∼48 hrs post surgery, the animals were sacrificed and the ME mucosa were surgically removed and divided into 0.5 mm^2^ square explants and individually seeded into a 24 well culture dish in media (75% DMEM, 25% HEMs-F12 supplemented with 5% bovine serum, and containing the following additives: 100 IU/mL penicillin, 100 mg/mL streptomycin, 0.4 mg/mL hydrocortisone and 10^−6^ M isoproterenol), as described in Palacios et al [29]. The explants were incubated in a 5% CO_2_ humidified atmosphere at 37°C. Culture media was replaced every 3 days. Photographs of each explant were taken daily with a RT-SPOT color digital camera to document the extent of primary culture growth. The diameter of explant outgrowth, which was approximately circular, was measured and its area was calculated using SPOT computer software calibrated to the magnification used.

### Immunostaining of rat middle ear tissue

Rat middle ears from control and NTHi treated animals were collected, fixed in 4% PFA and decalcified in 8% EDTA as previously described [30]. The ears were then placed in 30% sucrose in 0.1 M phosphate-buffered saline (PBS) for 1 day and then in OCT media for 1 hour under vacuum. They were subsequently mounted in OCT, snap frozen and processed to generate 10 μm cryosections. For immunohistochemistry, sections were incubated with 2% BSA and 15% normal goat serum in PBS pH 7.4 for 20 minutes to block non-specific binding followed by incubation with a chicken anti-Ecrg4 IgY (1.5 µg/mL) overnight at 4°C. The polyclonal IgY antibody was raised in chickens against recombinant human Ecrg4 (aa 71–148) and affinity purified by commercial contract with GenWay Biotech, Inc., (San Diego, CA). Purified pre-immune IgY from the same animal was used as a negative control. The following day, tissue sections were rinsed with PBS containing 0.3% Tween and incubated for 45 min at RT with goat anti-chicken antibody (1∶1000) labeled with Alexa Fluor 594 (Invitrogen, Carlsbad, CA) for immunofluorescent staining. Sections were then rinsed in PBS and covered with mounting media containing DAPI (Vector labs, Burlingame, CA). Nuclear and Ecrg4 staining was visualized by epifluorescent (Zeiss, Thornwood, NY) microscopy and photographed. In some experiments, sections were blocked then primary antibody applied overnight at 4°C as above but staining was visualized by light microscopy using DAB. The following day, sections were rinsed then incubated for 30 minutes with biotinylated donkey anti-chicken antibody at a 1∶1000 dilution (Jackson West Grove, PA). Sections were subsequently rinsed in PBS and stained with Vectastain Elite ABC kit (Vector Labs, Burlingame, CA). Finally, all specimens were lightly counter-stained with Hematoxylin (Richard-Allan Scientific, Kalamazoo, MI). Specificity of the primary antibody was determined by Western blotting with the same antibody as described below.

### Western blot

ME mucosal tissue from two Sprague-Dawley rats were surgically removed and whole cell extraction was carried out by suspending the tissue in 150 µL of T-PER lysis buffer (Pierce, Rockford, IL) supplemented with protease inhibitors (Roche, Indianapolis, IN), and sonicated briefly on ice. The concentration of proteins in the cell lysates was assessed using a BCA assay (Pierce), using BSA as a standard curve. Proteins (2 μg) were then separated by gel electrophoresis on 4–12% gradient NUPAGE gels (Invitrogen). The proteins were transferred electrophoretically onto polyvinylidene difluoride (PVDF) membranes using iBlot (Invitrogen, Carlsbad, CA). After blocking with 5% BSA in PBS-T, the membranes were incubated with chicken anti-Ecrg4 (Genway, San Diego, CA) at 1∶5000 dilution at 4°C overnight. The membranes were subsequently probed with horseradish peroxidase-conjugated anti-chicken (1∶10,000 dilution, Bio-Rad, Hercules, CA) at room temperature for one hour. Signals were detected using ECL-Plus kit (Pierce) according to the instructions of the manufacturer.

### Adenoviral and lentiviral constructs

Adenovirus (AD) encoding the open reading frame of the human Ecrg4 gene was originally prepared as described in Gonsalez et al. [27] using an AdEasy kit according to the manufacturer's recommendations (Agilent Technologies, La Jolla, CA). This adenoviral preparation was propagated from stock, purified and tittered using the Adeno-X^TM^ Maxi purification and the RapidTiter kits (Clontech, CA), respectively. The adenovirus expressing green fluorescent protein (ADgfp) was obtained from Vector Biolabs (Philadelphia, PA) and propagated according to the manufacturer's directions. To engineer the lentivirus plasmid constructs, the coding sequence of human Ecrg4 was PCR amplified from a commercial plasmid containing the full-length human cDNA (Origene, Rockville, MD). The sense primer, 5′-AGTCCTCGAGCCCCGCCGCCATGGC-3′, was designed to retain the original Kozak sequence, with an engineered XhoI restriction site. The anti-sense primer, 5′- ATTCGGATCCATGGTTAGTAGTCATCGTA-3′, carried a BamHI restriction site. The PCR products were purified and cloned into pLVX-IRES-ZsGreen1 (Clontech). The identity of the resulting plasmid, pLVX-IRES-ZsGreen1+Ecrg4, was confirmed by DNA sequencing (Retrogen, San Diego, CA). Lenti-ZsGreen+Ecrg4 viruses were packaged using a Lentiviral Packaging System (Clontech) then purified and tittered per the manufacturer's protocol.

### *In vitro* transductions

Explants were allowed to adhere to tissue culture wells for 48 hrs prior to transduction. After confirming that each explant had attached, the wells were divided randomly into three groups: (1) no treatment control, (2) ADgfp-treated, (3) ADEcrg4-treated. Each explant was treated overnight with media alone or media containing 10^5^ IU/mL of ADEcrg4, or 10^5^ IU/mL of ADgfp. The following morning, the wells were washed twice with ME media and then the explants were maintained in 350 µL of ME media and placed in a humidified incubator at 37°C under 5% CO_2_. The growth of the explants was then monitored by direct and/or fluorescent photography for 10 days following transduction.

For the lentivirus transfections, explants were produced as described above and allowed to adhere for 48 hrs prior to transfection. The plates were checked to confirm that the explants had attached. The wells of each 24-well plate were then divided randomly into three groups: Lenti-ZsGreen+Ecrg4 treated, Lenti-ZsGreen treated, and no treatment control. Each treatment group was transfected with 10^5^ IU/mL of lentivirus vector diluted in ME media. The no treatment control group received only ME media. The explants were incubated overnight with the lentivirus. The following morning the wells were washed twice with ME media and then the explants were allowed to grow in 350 µL of fresh ME media placed in a humidified incubator at 37°C under 5% CO_2_. The growth of the explants was monitored for 10 days following transduction.

### *In vivo* transductions

*In vivo* effects of ADEcrg4 and ADgfp were studied by injecting 50 µL of a 10^10^ IU/mL of virus (n = 6), trans-tympanically into the ME cavities of male Sprague-Dawley rats weighing ∼300 g. The tympanic membrane defects were sealed with small pieces of parafilm. The ears were examined two days later to exclude infection of the external or middle ears. Ears were then bilaterally inoculated with NTHi, biotype II as described above to induce ME infection. The NTHi control group received no pretreatment with adenovirus (n = 6). All the animals were sacrificed under general anesthesia 48 hrs after the NTHi infection corresponding to 96 hrs after the initial AD injection by intracardiac perfusion with PBS followed by 4% paraformaldehyde (PFA). The middle and inner ears were dissected intact and postfixed overnight and decalcified (in 8% EDTA and 4% PFA) for 14 days. The ME bullae were embedded in paraffin and sections were cut at 10 mm and H&E stained. ME sections were assessed for fluid area, number of inflammatory cells, and mucosal thickness as previously described to assess degree of ME inflammation [31], [32]. In brief, images of the same region from the largest area of the ME cavity taken at standardized location was used to calculate the percent area of the ME lumen occupied by inflammatory cells. Mucosal thickness was analyzed by computer-averaging the thickness of the epithelium at three standardized locations from these same sections. Data were obtained from three adjacent sections of six MEs in each condition.

### RNA extraction and RT-PCR

Total RNA was isolated from cells and tissues using TRIzol (Invitrogen). One mg total RNA from each sample was reverse transcribed using the iscript cDNA synthesis kit (Bio-Rad). Real-time PCR was performed to measure the expression of Ecrg4 mRNA using SYBR-green (Bio-Rad) and an IQ5 thermocycler according to the manufacturer's directions. The sequence for the sense primer was 5'-AAGCGTGCCAAACGACAGCTGTGGGAC-3', and for the antisense primer was 5'-TTAATAGTCATCATAGTTGACACTGGC-3'. The *gapdh* gene was used as a reference gene using the sense primer 5'-GCACAGTCAAGGCCGAGAAT-3', and antisense primer 5'-GCCTTCTCCATGGTGGTGAA-3'. Fold change was calculated using the comparative threshold cycle method [33]. Relative expression levels were normalized to *gapdh* gene and compared to either untreated or uninfected mucosa as indicated in the figure legends.

### Identification of transcription start sites by RACE

The SMARTer RACE Kit (Clontech) was used to analyze transcription start sites for the Ecrg4 gene following the manufacturer's protocol. The kit incorporates a universal primer during the reverse transcription and cDNA synthesis step. Following RNA extraction as described in the previous section, mRNA was isolated using Qiagen Oligotex mRNA kit per manufacturer protocol. The mRNA was then used to generate the cDNA. Ecrg4-specific transcripts were produced by nested PCR using the manufacturer's primers corresponding to the nested sequence and Ecrg4 gene-specific primers. The 3′ gene-specific primer (GSP) was: 5′-CATCATAGTTGACACTGGCTCCATGCCTG -3′ and the nested primer (NGSP) was: 5′-TAGCCAATAGTTGACATCATCT -3′. The amplified products were analyzed by 1% agarose gel electrophoresis, purified using QIAquick gel extraction kit and then cloned using the TOPO TA Cloning kit (Invitrogen). Ten clones were sequenced from each 5′ RACE PCR reaction.

### DNA microarrays

The profile of Ecrg4 gene expression in addition to several epithelial markers present in the mucosal epithelium during the course of OM inflammatory response in mice was evaluated using DNA microarrays as described elsewhere [32], [34]. In brief, 20 age-matched C57Bl/6:CB F1 hybrid mice purchased from Jackson Laboratories (Bar Harbor, ME) per time point were inoculated bilaterally with NTHi strain 3655 as described previously. Uninoculated, naive animals (time 0) served as controls. The ME mucosa were harvested and combined at each of the following post-infection intervals: 0 (no treatment), 3 hrs, 6 hrs, in addition to 1, 2, 3, 5 and 7 days after NTHi inoculation. Total RNA was extracted using TRIzol (Invitrogen). The quality of RNA was assessed using the RNA 6000 Labchip Kit on the Agilent 2100 Bioanalyzer to ensure the integrity of 18S and 28S ribosomal RNA. The mRNA was reverse transcribed using a T7-oligodT primer then transcribed using T7 RNA polymerase to generate biotinylated cRNA probes that were hybridized to two Affymetrix MU430 2.0 microarrays per time point sample. This procedure was then duplicated for each time point to obtain a second, independent replication.

### Statistical analysis

All transduction assays were performed at least three times in 24-cell culture plates under the indicated conditions. The outgrowth surface area was the Mean ± SEM for eight wells from one representative experiment. Statistical analysis was performed using one-way ANOVA, and differences were considered statistically significant at P<0.05.

## Results

### Ecrg4 is present in normal epithelial mucosal tissue

**Figure 1:**
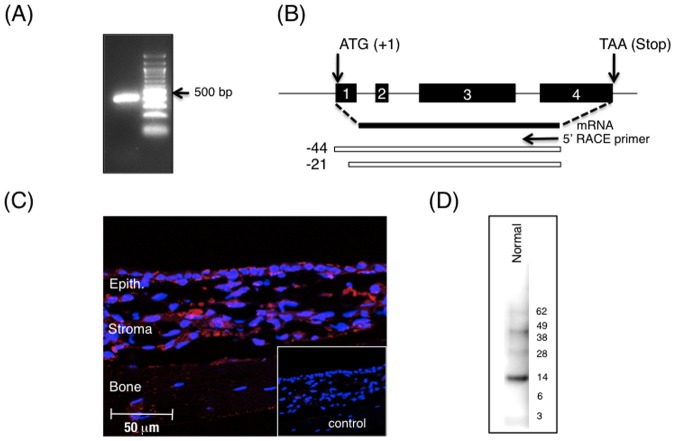
Ecrg4 is present in normal rat ME mucosa. **Panel A:** Ecrg4 PCR product amplified from uninfected rat ME mucosa cDNAs. A 100 bp molecular size marker is shown on the left lane. **Panel B:** Organization of the rat Ecrg4 gene shows the 4 exons and the start (set to +1) and stop codons of the open reading frame (ORF). The mRNA isolated from rat ME mucosa was subjected to 5′RACE analysis using a primer annealing to exon 4 (solid arrow). Following primer elongation, cloning and sequencing of the 5′ RACE products (clear bars), translation start sites (TSS) at −42 bp and −21 bp upstream of the ATG translation start site produce the identical ORF. An asterisk denotes that the −21 bp TSS was the most frequently amplified. **Panel C:** Immunolocalization of Ecrg4 (red) in the ME epithelium compared to a background immunofluorescence signal using pre-immune IgY (insert). Nuclei are counterstained with DAPI (blue). Epith.  =  epithelial cells (D) Immunoblotting of rat ME mucosa revealed the presence of a prominent 14 kDa Ecrg4 protein band.

We used PCR, 5′RACE (rapid amplification of cDNA ends), immunoblotting, and immunohistochemistry to demonstrate Ecrg4 gene expression and the presence of the Ecrg4 protein in normal rat ME mucosa. As shown in Figure 1A, PCR revealed the presence of the expected Ecrg4 product in normal epithelial mucosa. We also confirmed that there is one main transcript of the gene spanning exon 1 through exon 4. When 5′ RACE was deployed using total RNA (mRNA) prepared from the mucosa, a major transcription initiation site was detected and localized to −41 to −21 bp to the 5′ of the ATG translation start codon in the Ecrg4 open reading frame. Immunohistochemistry using an anti-Ecrg4 antibody demonstrated the presence of the protein in both the epithelium and stroma of normal mucosa (Figure 1B). The combination of granular and cell membrane localization suggests the presence of Ecrg4 in secretory granules [23], [35] and on the surface of the epithelium [14], [15]. Labeling of bone seen in Figure 1C was considered nonspecific. Finally, immunoblotting of mucosal lysates (Fig. 1C) revealed the presence of the expected ∼14 kDa Ecrg4 protein. Smaller peptide products predicted by post-translational processing [13], [14], [19], [20] were not detected.

### Down-regulation of Ecrg4 expression in epithelial mucosa after infection

**Figure 2:**
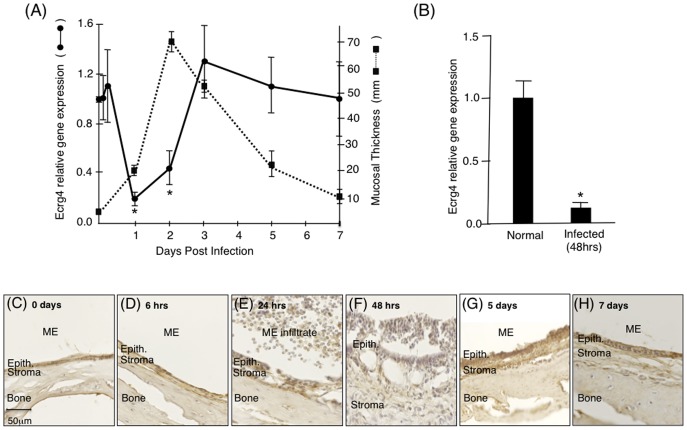
Characterization of changes in Ecrg4 gene expression in ME mucosal after NTHi infection. **Panel A:** Mining a genechip microarray showed time-dependent decreased Ecrg4 expression levels after NTHi infection (solid line) of mouse ME. The decrease was compared to the thickness of the ME mucosa (dashed line). Mouse Ecrg4 expression is down-regulated within 24 hrs, while mucosal hyperplasia increases beginning 24 hrs after infection and peaking at 48 hrs. Ecrg4 expression also recovers just prior to return of the mucosa to normal thickness. Each gene expression data point represents gene arrays obtained from 2 independent sets of 20 C57BL/6J mice and expressed as fold change from the expression levels measured at time 0 hr (see [32], [34] for details). *P<0.05. **Panel B:** RT-PCR confirmed that Ecrg4 mRNA is expressed in normal rat ME mucosa and that it is down-regulated 48 hrs after NTHi infection. Bars represent the mean ± SEM (n = 4 MEs per time point). *Significantly different from normal (P<0.05). **Panels C–H** Immunohistochemistry of rat ear tissue harvested at 0 hrs, 6 hrs, 24 hrs, 48 hrs, 5 days, and 7 days after NTHi infection showed changes in Ecrg4 immunostaining in the ME mucosa. Epith.  =  epithelial cells.

To investigate the role of Ecrg4 in mucosal epithelia, we turned to a well-described model of mucosal epithelial inflammation that is generated by bacterial infection of the ME. In this animal model, a robust and reproducible hyperplasia of the ME mucosa is induced by inoculation with NTHi. The mucosal response is minimal up to 12 hrs post-inoculation. However, at 24 hrs the mucosa begins to develop a pseudostratified, columnar epithelium with some ciliated and goblet cells, and an expanded stroma. By 48 hrs, mucosal thickness peaks, and the tissue then recovers over the next several days as OM resolves. The mucosal response is accompanied by a robust leukocytic infiltrate [29], [31], [36], [37], [38]. Mining a gene expression database generated by Affimetrix genechip analyses of control and infected mucosa of C57BL/6J:CB F1 mice [34], [39] established that mouse Ecrg4 gene expression was altered in the normal epithelial response to infection. As shown in Figure 2A (solid line), there was a dramatic down-regulation of Ecrg4 at 24 and 48 hrs post NTHi infection. This early decrease in Ecrg4 gene expression preceded the maximal increase in epithelial mucosal thickness observed at 48 hrs after NTHi infection (Figure 2A, hashed line). The observed Ecrg4 down-regulation was confirmed using qPCR at normal (pre-infected) and at 48 hrs post infection of rat MEs (Figure 2B). Immunohistochemistry of infected rat MEs (Figure 2C–H) showed analogous changes in Ecrg4 staining and mucosal morphology during the NTHi inflammatory response to those inferred from Figure 2A. Under normal conditions (Figure 2C), the epithelial mucosa appears as a thick layer attached to the ME bone with Ecrg4 staining. Over the first 24 hrs (Figure 2C–E), changes in immunostaining were apparent as the inflammatory response developed and the epithelial layer expanded, but overall Ecrg4 protein expression appeared decreased at 48 hrs (Figure 2F) when the epithelial mucosa thickness peaked. Ecrg4 staining recovered as mucosal thickness reversed and the infiltrating cells cleared over 5–7 days after infection (Figure 2G,H).

**Figure 3:**
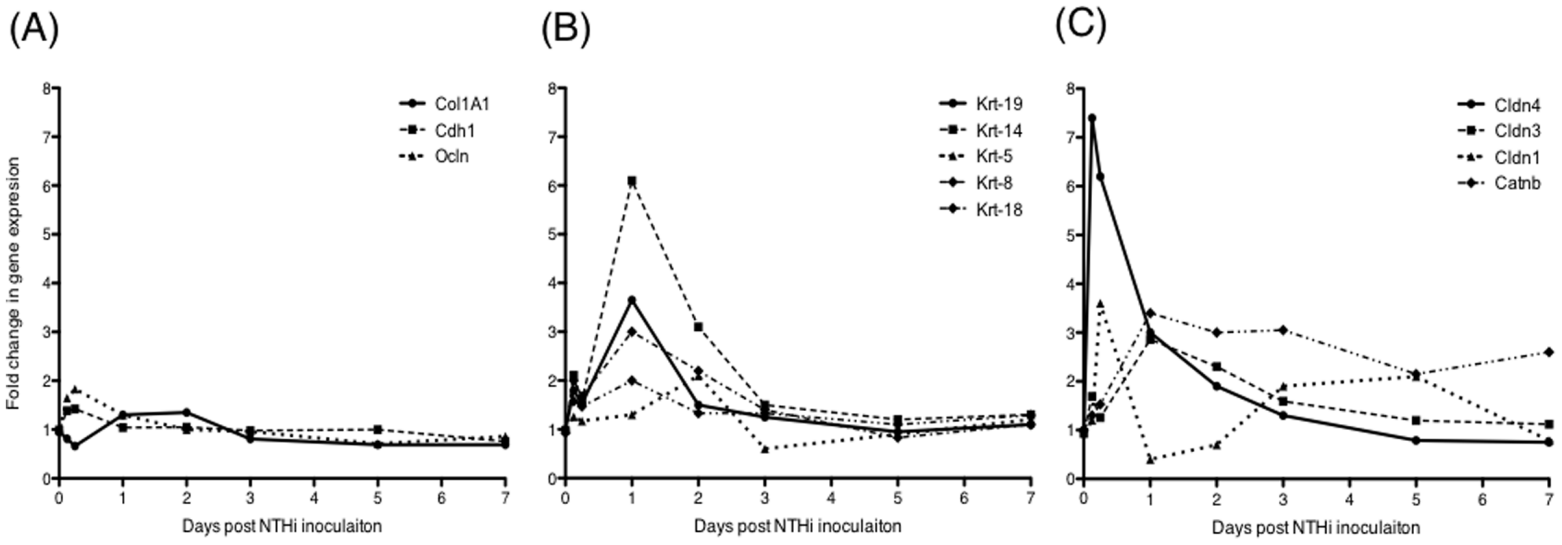
Changes in gene expression of epithelial cell genes in the responding mucosal epithelium after NTHi infection. Panel A, Unchanged. The expression of some epithelial genes like E-cadherin (Cdh1), occludin (Ocln) and collagen-1A1 (Col1A1) were minimally affected by the inflammatory response after NTHi infection. Panel B, Epithelial cytokeratins: The expression of several cytokeratins in the ME increased in correlation to the inflammatory response and mucosal thickening at 24 to 48 hrs. cytokeratin 19 (Krt-19), cytokeratin 14 (Krt-14), cytokeratin 18 (Krt-18), cytokeratin-5 (Krt-5) and cytokeratin 8 (Krt-8). Panel C, Tight junction genes: Expression of the claudins family that have been shown to specifically target tight junctions. Claudin-4 (Cldn-4) and claudin-1 (Cladn-1) gene expression was up-regulated early in response to NTHi infection. In contrast, changes in claudin-3 (Cladn-3) and β-catenin gene expression were moderate and up-regulated later on in the course of OM.

To further explore the relationship between Ecrg4 expression and the mucosal epithelium, we analyzed the DNA microarray data for well-characterized epithelial markers [40] and evaluated their expression levels at different times after infection. The gene expression of twelve commonly used epithelial genes is shown in Figure 3, and the ranges and statistics are provided in Table S1. These markers were selected for encoding either filament forming proteins like cytokeratins (Krt), and collagen (Col1A1) which define the cytoskeleton structure and serve a mechanical support role, or alternatively intercellular tight junctions and junction protein markers like E-cadherin (Cdh-1), β-catenin (Catnb), occludin (Ocln), and the claudins (Cldn), which participate in epithelial barrier function.

As shown in Figure 3A, the expression levels of some epithelial marker genes including E-cadherin, occludin and collagen1A1 showed minimal change during OM (Figure 2A). In contrast, the structural cytokeratin genes (Figure 3B), showed increases peaking at 24 hrs with cytokeratins-8, -14, and -19 being the most highly regulated at times when the epithelium is expanding. Junctional proteins showed either early up-regulation (claudins-1 and -4), or up-regulation at 3–6 hrs (Figure 3C), or at 24 hrs (claudin-3, and β-catenin) which slowly decreased to normal. These results indicate that epithelial histopathology during OM correlates positively with the expression kinetics of several epithelial genes. They also provide additional evidence that the down-regulation of Ecrg4 during OM is not the result of epithelial damage.

### Ecrg4 over-expression *in vitro* inhibits mucosal epithelial cells

**Figure 4:**
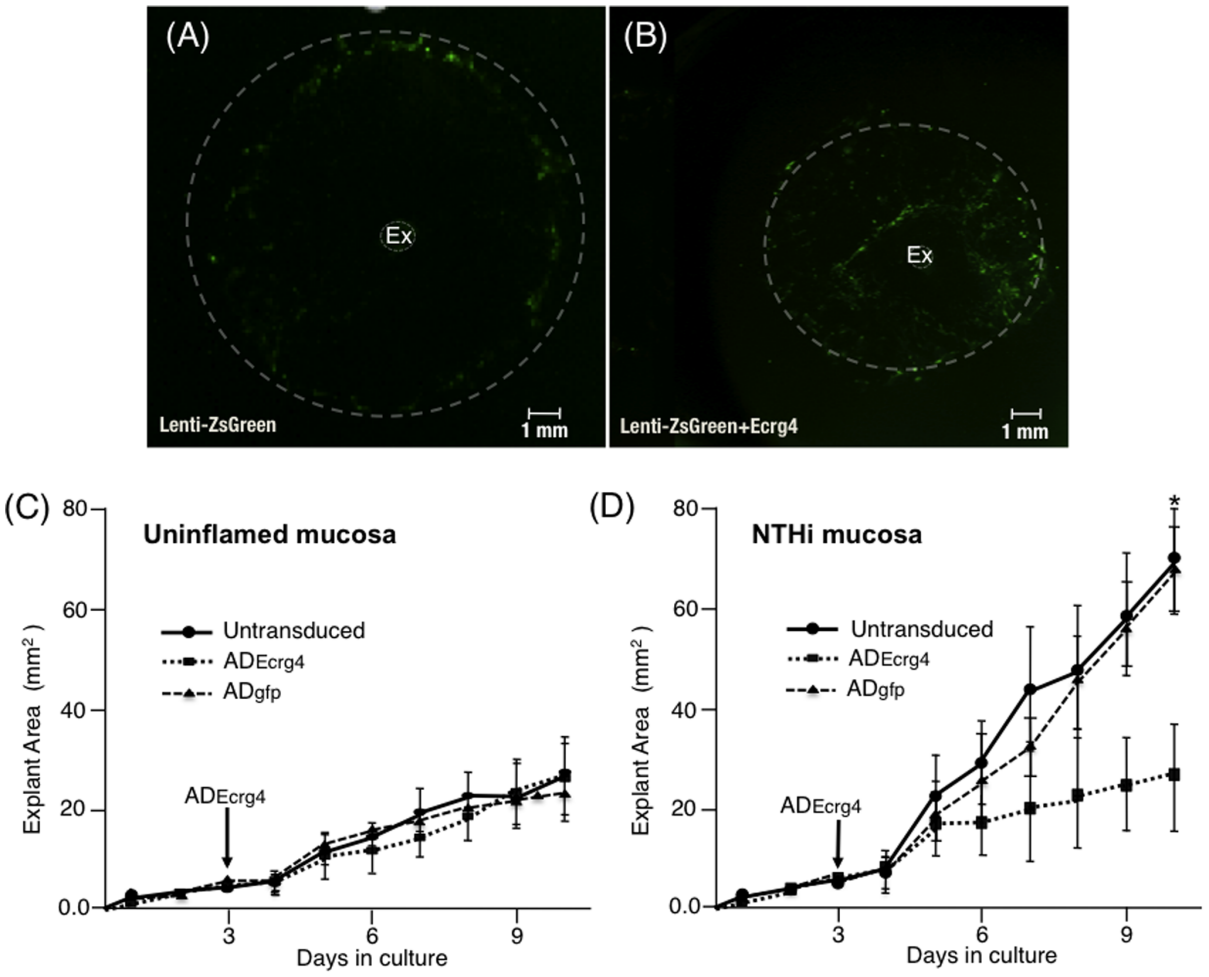
The effect of Ecrg4 expression on mucosal epithelial growth *in vitro*. **Panel A:** Fluorescent images of representative NTHi-infected ME explant outgrowth after transduction with Lenti-ZsGreen or **(Panel B)** lenti-ZsGreen+Ecrg4 and culture for 10 days. The NTHi-induced hyperplastic growth response is decreased by Ecrg4 gene expression *in vitro* suggesting that Ecrg4 is a regulatory component of the cellular response to inflammation. Explants of mucosa from MEs were harvested and cultured *in vitro* for 2 days then transduced. **Panel C:** Surface area quantification of control (uninfected) explant expansion showing no effect of transduction with ADEcrg4. **Panel D:** ADEcrg4 transduction dramatically decreases the growth of mucosal explants harvested from NTHi-infected MEs, when compared to those transduced with ADgfp or non-transduced infected explants. In panels C and D, n>6 explants per group per time point, bars represent mean ± SEM with *P<0.05.

Our expression data indicated that Ecrg4 down-regulation might play a role in regulating mucosal hyperplasia. To test this hypothesis, we used a transduction strategy utilized to explore the possibility that the natural inhibition of Ecrg4 expression after infection was related to the hyperplasia of epithelial mucosa. Inflamed mucosa were harvested 48 hrs after NTHi infection, and explants placed into culture as described in Materials and Methods. Two days later, proliferating cells (>90% epithelial) were transduced with lentivirus [41] overnight. The resulting transduced cells expressed GFP (Lenti-ZsGreen) alone or both GFP and Ecrg4 (Lenti-ZsGreen+Ecrg4) in a bicistronic cassette. In both instances, GFP in the explants was detected by fluorescence microscopy and, as shown in Figure 4A, a differential distribution of GFP-fluorescence emerged. Lenti-ZsGreen transduced cells were primarily localized to the margins of the expanding explant (hashed lines), which migrated after transduction of the explant. In contrast, GFP-positive cells co-expressing Ecrg4 (Lenti-ZsGreen+Ecrg4), seen in Figure 4B, were found closer to the explant and the overall margins closer to the original explant than seen with GFP transduction alone. These results pointed to an inhibition of migration by Ecrg4.

To further investigate this observation and better understand the consequence of the natural decrease in Ecrg4 gene expression after infection *in vivo*, we quantified the inflammatory response ex vivo [29] and evaluated the effects of over-expressing the Ecrg4 gene, through an adenoviral (AD) transfection (Figure 3C,D). Epithelial explants from control (uninfected) or NTHi-infected (inflamed) ME mucosa were cultured as previously described [38] and the effects of ADgfp or ADEcrg4 transduction on ME explant outgrowth quantified. As shown in Figure 4C, transduction of uninfected (control) explants with ADEcrg4 had no effect on explant expansion. Both untreated and ADEcrg4-transduced control explants grew at a rate of ∼2.0 mm^2^/day. In contrast, mucosal explants derived from NTHi-inflamed mucosa expanded at a rate of 6.5 mm^2^/day (Figure 4D solid line). When these explants were transduced with ADEcrg4, their expansion rate was reduced to 1.8 mm^2^/day (Figure 4D hashed line), a rate indistinguishable to that observed from both uninflamed (control) and untreated explants (Figure 4C) [30], [42]. Thus, Ecrg4 eliminated the effects of NTHi infection (Figure 4D) but had no effect on basal growth (Figure 4C). In the infected explants, expansion rates began to segregate 2 days after ADEcrg4 transduction, and remained decreased through the 11 days of the experiment. These data are consistent with the 24–48 hrs required for the accumulation of protein after foreign gene expression with AD transduction. Ecrg4 protein production and mRNA expression in the transduced explants were confirmed by immunoblotting and RT-PCR (not shown).

### Ecrg4 gain of function inhibits the *in vivo* inflammatory response to NTHi infection

**Figure 5:**
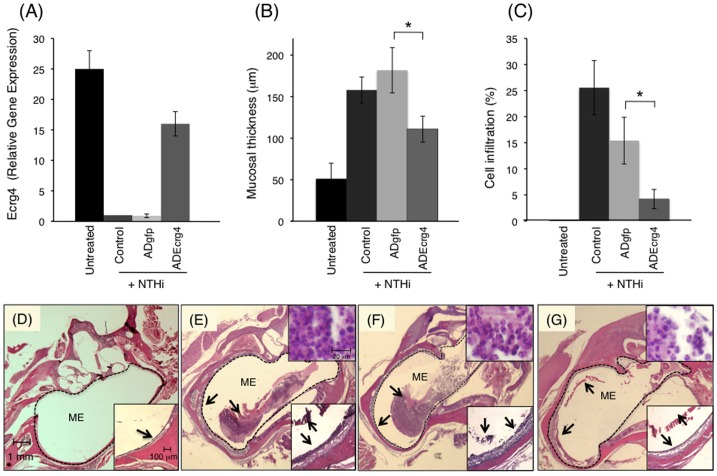
The impact of Ecrg4 over-expression on ME inflammation after NTHi infection *in vivo*. **Panel A:** RT-PCR shows the relative Ecrg4 gene expression in ME mucosal tissue 4 days after AD injection into the ME *in vivo* and 48 hrs post-infection. Untreated animals received no AD or NTHi injection (n = 4 ears) while control animals received NTHi with no follow-up AD injection. **Panel B:** Mucosal thickness measured in untreated animals (untreated) or 48 hrs after they received NTHi only (control), or NTHi after ADgfp (ADgfp + NTHi) or ADEcrg4 (ADEcrg4 + NTHi). Results are expressed as means ± SEM of six MEs, sampled at standardized locations. **Panel C:** Area of the ME cavity occupied by inflammatory cells was used to quantify leukocyte infiltration (n = 6 ears). Leukocytes were substantially reduced with Ecrg4 transduction. **Panels D–G:** Representative histological sections at 48 hrs post-NTHi challenge showing the MEs of untreated (**Panel D**), NTHi (**Panel E**), ADgfp + NTHi (**Panel F**), and ADEcrg4 + NTHi (**Panel G**) in rats. There is considerable inflammation in the ME space filling the cavity with fluid and inflammatory cells in the NTHi and ADgfp + NTHi groups. ME mucosa thickening is seen at a higher magnification in the inserts. Arrows denote the epithelial mucosa (**Panels D–G**) and mucosa and the inflammatory infiltrate (**Panels E–G**) within the ME space. Scale bars indicate magnification.

In order to investigate the physiological significance of the natural course of decreased Ecrg4 gene expression during inflammation *in vivo* (see Figure 1), we evaluated the effects of preventing the decrease by pre-injecting the ME with an ADEcrg4 that drives gene expression using a constitutive CMV promoter (Figure 2A). As shown in Figure 5, we first established that the pre-injection of ADEcrg4 onto the mucosal bed *in vivo*, two days before NTHi infection circumvented the loss of Ecrg4 protein expression which is normally observed 48 hrs after infection (Figure 5A). Whereas the infection of control or ADgfp animals resulted in a >90% decrease in mucosal Ecrg4 gene expression when compared to untreated animals (Figure 4A), Ecrg4 mRNA remained high in animals pre-injected with ADEcrg4 in spite of the NTHi infection (25 vs. 15 fold). These data demonstrated that the natural down-regulation of Ecrg4 expression observed during inflammation could be overcome by pre-injection of ADEcrg4, and that AD itself had no effect.

We then quantified the effects of preventing Ecrg4 down-regulation on mucosal thickness (Figure 5B) and cell infiltration (Figure 5C) in either (1) untreated animals (Untreated), or after (2) NTHi infection without AD (Control + NTHi), (3) NTHi infection after ADgfp pretreatment animals (ADgfp + NTHi), and (4) NTHi infection after ADEcrg4 pretreatment (ADEcrg4 + NTHi). Analyses of histological sections revealed that pretreatment with ADEcrg4 attenuated mucosal thickness (Figure 5B) and inhibited the inflammatory cell infiltration (Figure 5C) observed in this model [38], [43]. As illustrated in representative tissue sections, NTHi-injection led to a three-fold increase in mucosal thickness 48 hrs post-NTHi infection (Figure 5D,E). This response was unaffected by a pre-injection with ADgfp (Figure 5E,F), but there was a significant reduction in mucosal thickness in ADEcrg4-treated animals (Figure 5G). Similarly, there was extensive inflammatory cell infiltration present 48 hrs after NTHi injection in the control and ADgfp treated groups (Figure 5E,F) compared to untreated animals (Figure 5D). The group pretreated with ADEcrg4, however (Figure 5G), exhibited significantly fewer inflammatory cells in the ME cavity in spite of NTHi infection.

## Discussion

We used a well-characterized animal model of infection induced mucosal inflammation and hyperplasia [38], [43] to characterize the effects of Ecrg4 on mucosal cell migration and proliferation during infection. We found that Ecrg4 is constitutively localized to the normal mucosa but that unlike may other genes, Ecrg4 gene expression is rapidly down-regulated after infection. Histological and gene expression data indicate that the down-regulation is not secondary to loss of epithelial cells. The expression data are consistent with a role for Ecrg4 in regulating mucosal hyperplasia, but do not provide a test of this hypothesis. However, we have demonstrated that preventing this down-regulation by over-expression of Ecrg4 *in vitro* and *in vivo* altered the natural course of the inflammatory response by decreasing the outgrowth of epithelial cells from mucosal explants. We further observed that Ecrg4 over-expression had a profound effect on the ability of infiltrating inflammatory cells to migrate into the ME *in vivo*, indicating that Ecrg4 may play a role in mucosal epithelial barrier integrity. Together, these data suggest that the constitutive basal expression of Ecrg4 in mucosa may serve to maintain normal epithelial barrier function while inhibiting both hyperplasia and recruitment of inflammatory cells.

The ME infection model of mucosal hyperplasia has numerous advantages over other experimental models of mucosal epithelial barriers. First, it is an experimental model that is amenable to a localized rather then systemic infection. Second, this OM model induces a highly predictable and reproducible inflammatory response that enables quantitative analyses of inflammation *in vitro* and *in vivo* while allowing the use of normal and transduced tissues to study the effects of gained function. Finally, the OM model has been thoroughly characterized in terms of changes in global gene expression after infection [39], [43], [44] and, it includes a reversible, self-resolving component of inflammatory cell infiltration that, like epithelial hyperplasia, is quantifiable. Whether the results presented here translate to other epithelial mucosal barriers remains to be established and is currently under investigation. Interestingly, Ecrg4 is readily and similarly detected in epithelia of CNS [15], [27], [45], skin [16], gut, esophagus, lung, reproductive tract and skin (unpublished).

The fact that normal mucosa expresses Ecrg4 constitutively, but that its expression decreases after infection, is consistent with the hypothesis that Ecrg4 serves a sentinel function on the epithelial cell surface and senses the response to inflammation [13], [14]. Moreover, preventing the Ecrg4 decrease after infection affected both mucosal epithelial expansion (Figure 4) and leukocyte infiltration (Figure 5) through the mucosa and into the ME. This raises the possibility that constitutive Ecrg4 expression in mucosal epithelium regulates epithelial barrier integrity.

Recent experiments in our laboratories used Ecrg4 transduced epithelial cells in culture to establish that, while the mature 14 kDa Ecrg4 protein is secreted, it remains tethered to the cell surface [14]. It is presumed that this form of Ecrg4 has the inhibitory activity detected in the experiments, as described here. It is interesting to speculate that there is a link between the amounts of Ecrg4 protein displayed on the cell surface and the ability of epithelial cells to respond to inflammation. If so, the findings reported here would suggest that the capacity of gene expression to restore Ecrg4 protein onto the cell surface might define the extent and length of the inflammatory response to infection.

While the data presented here establish local Ecrg4 expression in mucosal epithelia, previous studies have shown that Ecrg4 is like many other cytokines and chemokines in that it can be expressed in leukocytes. For example, Matsuzaki et al. [35] detected Ecrg4 in T-cell lines and suggested that it plays an anti-apoptotic role in activated T-cells. We have previously demonstrated the presence of Ecrg4 on the surface of leukocytes and shown that it is shed from the cell surface with cell activation by LPS [13]. Taken together, these data coupled with our current observation in the reduction of inflammatory cell infiltration of the ME cavity again support the hypothesis that Ecrg4 participates in the inflammatory cascade that follows infection.

On a final note, Ecrg4 gene expression is epigenetically regulated by methylation of its promoter, and through hypermethylation, its down-regulation is closely associated with epithelial cancer development and metastases [26], [], [28,46]. The results here add to a growing body of evidence that Ecrg4 gene expression is linked to the inflammation, infection and the injury response [13], [14], [15], [16], [27]. If an epigenetic set point controlling Ecrg4 expression and regulating Ecrg4 activity in normal tissues exists, then the results here suggest that therapeutics targeting Ecrg4 may modulate mucosal epithelial resistance to infection.

## Supporting Information

Table S1 **Overview of epithelial markers gene expression after NTHi inoculation.** A survey of several well characterized epithelial markers showed that there was no down-regulation of any of these genes in the middle ear mucosa in correlation to Ecrg4 gene expression during the same time course.
(DOCX)
