# Supplementary material for: Ecrg4 Attenuates the Inflammatory Proliferative Response of Mucosal Epithelial Cells to Infection
Source: PLoS One. 2013 Apr 23;8(4):e61394. doi: 10.1371/journal.pone.0061394 (PMC3634077; doi:10.1371/journal.pone.0061394)
Supplement: Table S1 — Overview of epithelial markers gene expression after NTHi inoculation. A survey of several well characterized epithelial markers showed that there was no down-regulation of any of these genes in the middle ear mucosa in correlation to Ecrg4 gene expression during the same time course. (DOCX) [file pone.0061394.s001.docx]

**Table S1. Overview of epithelial markers gene expression after NTHi inoculation. A survey of several well characterized epithelial markers showed that there was no down-regulation of any of these genes in the middle ear mucosa in correlation to Ecrg4 gene expression during the same time course.**

| Time: | 0h | 3h | 6h | 24h | 2d | 3d | 5d | 7d |
| --- | --- | --- | --- | --- | --- | --- | --- | --- |
| **E-cadherin (Cdh1)** | | | | | | | | |
| Fold Exp | 0.993 | 1.386 | 1.424 | 1.042 | 1.045 | 0.98 | 1.023 | 0.76 |
| Range | 0.80-1.10 | 1.20-1.50 | 1.30-1.56 | 0.99-1.10 | 0.95-1.14 | 0.78-1.23 | 0.93-1.13 | 0.62-0.93 |
| P-Value | 0.96 | 0.21 | 0.16 | 0.57 | 0.71 | 0.95 | 0.86 | 0.41 |
| **Beta-catenin (Catnb)** | | | | | | | | |
| Fold Exp | 1.0 | 1.29 | 1.53 | 3.39 | 2.97 | 3.05 | 2.15 | 2.60 |
| Range | 0.9-1.01 | 1.21-1.37 | 1.50-1.55 | 3.1-3.7 | 2.8-3.2 | 2.90-3.2 | 2.0-2.35 | 2.4-2.81 |
| P-Value | 0.998 | 0.15 | 0.02 | 0.05 | 0.05 | 0.02 | 0.07 | 0.05 |
| Cytokeratin 19 (Krt1-19) | | | | | | | | |
| Fold Exp | 0.99 | 1.81 | 1.53 | 3.65 | 1.49 | 1.26 | 0.94 | 1.11 |
| Range | 0.86-1.14 | 1.6-1.99 | 1.20-2.10 | 2.88-4.6 | 1.10-1.90 | 1.00-1.58 | 0.91-0.99 | 1.00-1.20 |
| P-Value | 0.96 | 0.10 | 0.4 | 0.11 | 0.37 | 0.49 | 0.45 | 0.34 |
| Cytokertain 14 (Krt1-14) | | | | | | | | |
| Fold Exp | 0.982 | 2.11 | 1.58 | 6.07 | 3.17 | 1.50 | 1.19 | 1.29 |
| Range | 0.81-1.19 | 1.99-2.23 | 1.14-2.18 | 5.09-7.23 | 3.17-3.16 | 1.47-1.52 | 1.13-1.26 | 1.19-1.41 |
| P-Value | 0.94 | 0.05 | 0.39 | 0.06 | 8e-5 | 0.03 | 0.19 | 0.21 |
| **Cytokeratin 5 (Krt1-5)** | | | | | | | | |
| Fold Exp | 0.79 | 1.26 | 1.17 | 1.30 | 2.12 | 0.59 | 0.91 | 1.20 |
| Range | 0.39-1.61 | 1.18-1.34 | 0.81-1.70 | 0.93-1.80 | 1.71-2.62 | 0.20-1.73 | 0.66-1.24 | 0.65-2.20 |
| P-Value | 0.80 | 0.17 | 0.74 | 0.57 | 0.18 | 0.71 | 0.81 | 0.82 |
| Cytokeratin 8 (Krt2-8) | | | | | | | | |
| Fold Exp | 0.93 | 2.00 | 1.68 | 3.05 | 2.20 | 1.44 | 0.83 | 1.14 |
| Range | 0.64-1.36 | 1.76 -2.27 | 1.62 -1.75 | 2.33-4.0 | 1.77- 2.75 | 1.35 -1.53 | 0.68 -1.01 | 0.91-1.44 |
| P-Value | 0.88 | 0.11 | 0.04 | 0.15 | 0.17 | 0.11 | 0.5 | 0.67 |
| Claudin 4 (Cldn4) | | | | | | | | |
| Fold Exp | 1.0 | 7.3 | 6.17 | 2.98 | 1.87 | 1.31 | 0.79 | 0.75 |
| Range | 0.94-1.06 | 7.12-7.64 | 5.33-7.15 | 2.77-3.21 | 1.75-2.00 | 1.22-1.41 | 0.78-0.80 | 0.73-0.76 |
| P-Value | 0.98 | 0.010 | 0.05 | 0.04 | 0.07 | 0.17 | 0.03 | 0.05 |
| **Claudin 3 (Cldn3)** | | | | | | | | |
| Fold Exp | 1.0 | 1.51 | 2.145 | 3.89 | 12.3 | 1.97 | 1.54 | 0.89 |
| Range | 0.99-1.10 | 0.99-2.31 | 1.23-3.74 | 2.54-5.96 | 7.29-20.76 | 1.30-3.00 | 1.12-2.11 | 0.61-1.30 |
| P-Value | 0.98 | 0.50 | 0.40 | 0.19 | 0.13 | 0.35 | 0.40 | 0.80 |
| **Claudin 1 (cldn1)** | | | | | | | | |
| Fold Exp | 0.93 | 1.22 | 3.65 | 0.39 | 0.73 | 1.89 | 2.14 | 0.79 |
| Range | 0.63-1.36 | 1.05-1.40 | 2.50-5.39 | 0.35-0.44 | 0.41-1.32 | 0.87-4.11 | 1.62-2.83 | 0.60-1.04 |
| P-Value | 0.88 | 0.40 | 0.19 | 0.08 | 0.69 | 0.56 | 0.22 | 0.55 |
| Occludin (Ocln) | | | | | | | | |
| Fold Exp | 0.993 | 1.65 | 1.84 | 1.30 | 1.02 | 0.96 | 0.71 | 0.86 |
| Range | 0.88-1.1 | 1.40-1.96 | 1.32-2.54 | 1.20-1.40 | 0.91-1.13 | 0.90-1.03 | 0.70-0.73 | 0.80-0.99 |
| P-Value | 0.96 | 0.21 | 0.31 | 0.18 | 0.90 | 0.68 | 0.05 | 0.48 |
| Procollagen-1 (ColA1) | | | | | | | | |
| Fold Exp | 0.954 | 0.811 | 0.66 | 1.31 | 1.35 | 0.81 | 0.69 | 0.69 |
| Range | 0.7-1.3 | 0.55-1.19 | 0.6-0.8 | 1.1-1.61 | 0.59-3.1 | 0.75-0.86 | 0.64-0.74 | 0.59-0.81 |
| P-Value | 0.90 | 0.68 | 0.27 | 0.40 | 0.78 | 0.20 | 0.13 | 0.258 |
